# Mortality Rate of Infection With COVID-19 in Korea From the Perspective of Underlying Disease

**DOI:** 10.1017/dmp.2020.60

**Published:** 2020-03-31

**Authors:** Yun-Jung Kang

**Affiliations:** Department of Clinical Laboratory Science, Sang-ji University, Wonju, Korea

**Keywords:** COVID-19, mortality rate, over 70, perspective, prevention, underlying diseases

## Abstract

On December 31, 2019 the China National Health Commission (NHC) reported that an unknown cause of pneumonia had been detected in Wuhan in Hubei province. On February 12, the disease caused by the novel coronavirus (2019-nCoV) was given a formal name, COVID-19. On January 20, 2020, the first case of COVID-19 was confirmed in Korea. The age-specific death rate was the highest among patients over 70 years of age, with underlying diseases in their circulatory system, such as myocardial infarction, cerebral infraction, arrythmia, and hypertension. Patients with underlying disease who are 70 years of age or older should recognize that there is a high possibility of developing a serious disease in case of viral infection and follow strict precautions.

On December 31, 2019, the China National Health Commission (NHC) reported that an unknown cause of pneumonia had been detected in Wuhan in Hubei province. The NHC later confirmed that the infection was a novel coronavirus-infected pneumonia (NCIP). On February 12, the disease caused by the novel coronavirus (2019-nCoV) was given a formal name, COVID-19. On January 20, 2020, the first case of COVID-19 was confirmed in Korea.^1^ After the first COVID-19 case was confirmed on January 20, 2020, the Korea Centers for Disease Control and Prevention has focused on delaying the inflow of the virus into Korea and its spread in local communities, with considerable success. Also, the mortality rate of COVID-19 outside China was rather low, leading to the expectation that the disease’s impact on national health would be minor. However, things turned over rapidly after Case No. 31. The epidemic is spreading rapidly on a global level.^2^ The Secretary General of World Health Organization, Tedros Adhanom Ghebreyesus, warned that the COVID-19 epidemic has reached its watershed and that every state should prepare for it.^3^

Among the symptoms of COVID-19 are fever and minor respiratory symptoms, such as dry coughs, which overlap with other respiratory diseases; therefore, it is not easy to confirm a case based only on early symptoms. When the symptoms get worse and lead to serious respiratory symptoms, such as dyspnea, low oxygen saturation, and pneumonia, they can cause death.^4^

The current situation in Korea regarding COVID-19 occurrences at 00:00, March 16, 2020, is as follows: the total number of confirmed cases are 8236, with 1137 released from quarantine among them. New confirmed cases are counted to be 74, while the total number of deaths is 75. Most of the dead had underlying diseases or were elderly (Table 1). The mortality rate is 1-2% but cannot be confirmed yet. Among the deaths, age-specific death rate was highest among patients over 70 years of age, with underlying diseases in their circulatory system, such as myocardial infraction, cerebral infraction, arrythmia, and hypertension. By the term “underlying diseases,” we refer to chronic diseases of the patient, such as hypertension, diabetes, asthma, renal failure, and tuberculosis.^5^

**TABLE 1:** Status and Characteristics of Domestic Deaths (00:00, March 16th)

**Category**		**Persons (** ***n*** **)**	**Rate (%)**	**Remarks**
Deaths	–	75		Mortality rate (out of 8236 confirmed patients) 0.91%
Mortality rate by age	30s (849 confirmed)	1	0.12	
	40s (1147 confirmed)	1	0.09	
	50s (1585 confirmed)	6	0.38	
	60s (1024 confirmed)	14	1.37	
	70s (531 confirmed)	28	5.27	
	80s ≤ (270 confirmed)	25	9.26	
Underlying disease (can be duplicated)	Circulatory system disease	47	62.7	Myocardial infarction, cerebral infarction, arrhythmia, hypertension, etc.
	Endocrine and metabolic diseases	35	46.7	Diabetes, hypothyroidism, etc.
	Mental illness	19	25.3	Dementia, schizophrenia, etc.
	Respiratory diseases	18	24.0	Asthma, chronic obstructive pulmonary disease, pneumonia, etc.
	Urinary and reproductive system diseases	11	14.7	
	Malignant neoplasm (cancer)	10	13.3	
	Nervous system diseases, etc.	3	4.0	
	Digestive system diseases	2	2.7	
	Blood and hematopoietic diseases	1	1.3	
High risk group	65 y and older	61	81.3	
	underlying disease(o)	74	98.7	

[source] Korea Centers for Disease Control and Prevention.

Korea had its first case of swine flu in May 2009, with the first death on August 15 the same year. After that, on October 26, the government announced children under 59 mo old, pregnant women, and mothers within 2 wk of delivery, citizens over 65 y old, patients with chronic lung diseases, chronic cardiovascular diseases (except for hypertension), diabetes, chronic renal diseases, chronic liver diseases, cancers, people with weakened immunity, and other patients with absorption risks as high risk group to the complications of the swine flu and recommended them treatment in time, according to its 6th version of the Guidelines for Preventing and Managing Swine Flu.^6^

Currently, the Korea Centers for Disease Control and Prevention is planning to categorize patients according to their pulse, age, and underlying diseases they had upon being found infected; the Centers would transfer critical patients to negative pressure isolation rooms designated by the government for proper treatment. The Centers came up with this new plan in the situation where the number of confirmed cases and death increased rapidly, leading to the importance of judging and categorizing the seriousness of patients’ situation.^7^

The Korean Diabetes Association argued that patients who are suffering underlying diseases with high risk of death should be given access to early diagnosis and treatment of COVID-19. It requested preferential opportunities of examination and hospitalization for people over 70 with diabetes when they had suspected symptoms. According to a recent research article on Chinese patients, which was published in *The Journal of the American Medical Association*, the overall death rate was 2.3% among 44,672 patients; however, the mortality rate leaped to 8.0% in people in their 70s and 14.8% in people in their 80s. Patients with diabetes also showed higher mortality rate of 7.3%.^8^

Even when they are exposed to the virus on the same conditions, people with underlying diseases should be aware that they are more susceptible to infection than people without them, as they have weaker immunities; they must adhere to the prevention regulation strictly. Especially, citizens over 70 with underlying diseases should be classified as high-risk group and managed carefully. This research would be provided as a basic material for guidelines regarding disease prevention and management of high-risk group among confirmed cases for future infectious diseases.

## Data Availability

The datasets used and/or analyzed during the current study are available from the corresponding author on reasonable request.
